# The first magnetically controlled growing rod (MCGR) in the world – lessons learned and how the identified complications helped to develop the implant in the past decade: case report

**DOI:** 10.1186/s12891-021-04181-0

**Published:** 2021-04-01

**Authors:** Jason Pui Yin Cheung, Kam Yim Sze, Kenneth Man Chee Cheung, Teng Zhang

**Affiliations:** 1 Department of Orthopaedics and Traumatology, The University of Hong Kong, Pokfulam, Hong Kong, SAR China; 2 Department of Mechanical Engineering, The University of Hong Kong, Pokfulam, Hong Kong, SAR China

**Keywords:** Magnetically controlled growing rod, MCGR, Distraction failure, Rotor stalling, Metallosis, Crooked rod

## Abstract

**Background:**

The first magnetically controlled growing rod (MCGR) was implanted in 2009. Since then multiple complications have been identified that have helped drive the development of the MCGR and its surgery. The aim of this report is to illustrate how identified complications in the first MCGR helped with developments in the past decade and to report a unique failure mechanism with stud fracture close to the barrel opening.

**Case presentation:**

A 5-year old girl with a scoliosis of 58.5 degrees at T1–9 and 72.8 degrees at T9-L4 had a single MCGR inserted and anchored at T3–4 and L3–4. At postoperative 13 months the MCGR was noted to have lost of distraction between lengthening episodes due to unrestricted turning of the internal magnet. To prevent further loss of distraction, an external magnet was placed outside the skin to prevent the magnet from turning back. The overall balance was suboptimal and after the rod was fully distracted, proximal junctional kyphosis occurred. Subsequently, the MCGR was modified with an internal keeper plate to prevent loss of distraction and a dual set of these rods were implanted when the patient was 9 years old. Extension proximally to C7-T1 was done to manage the proximal junctional kyphosis. Her spinal balance improved and distractions continued. She subsequently developed add-on below and the piston rod was not aligned with the actuator. The lumbar spine was also observed to have autofusion. She subsequently had final fusion surgery performed at the age of 15 from C7-L4 leaving a residual tilt below to avoid fusion to the pelvis. The final extracted rod on the left side indicated the “crooked rod sign” on X-ray and rod dissections revealed a new failure mechanism of stud fracture close to the barrel opening. Body fluids and tissue may infiltrate the rod despite no obvious deformation or fractures resulting in hastened wearing of the threads.

**Conclusions:**

There are various complications associated with MCGRs that are related to rod design and surgical inexperience. Repeated rod stalling is not recommended with potential stud fracture and “crooked rod sign”. Rotor stalling and thread wearing which indicates rod failure still require solutions.

## Background

Growing rods are now the standard treatment option for EOS because it prevents curve deterioration while accomodating physiological spinal growth [1–3]. Traditional growing rods require surgical distractions usually every 6 months [1, 2, 4–8] but carries significant risk of anesthestic and wound complications [9]. The magnetically controlled growing rod (MCGR) has revolutionized surgery for EOS as it allows gradual outpatient lengthening [10–13]. This avoids the risks with repeated surgical lengthening [1, 14, 15] and allows more frequent distractions to mimic physiological growth [16]. Regular distractions may also prevent autofusion associated with the traditional growing rod (TGR) in which abrupt and forceful open surgical distractions are imposed [15, 17]. It has been extended to treating patients with thoracic insufficiency syndrome [18] and gradual correction of severe deformities [19, 20]. MCGRs have obvious advantages with awake distractions and avoids repeated admissions with surgery under general anesthesia. The MCGR also has cost-saving benefits over TGR [21, 22].

However, complications of MCGR are not uncommon. There are reports of unplanned reoperations of up to 46.7% of patients at 2-year follow-up [23]. Of these complications, the most common reasons for revision surgery are failure of rod distraction and proximal foundation failure. Distraction failure can be technical or mechanical in origin. Technical complications are usually avoidable and include inappropriate rod bending, and incorrect rod insertion and configuration. This leads to failures of distraction along the long axis of the rod. Mechanical failures include spontaneous bone formation near the housing unit, actuator pin fracture [24], and rod stalling/clunking. All of these lead to limitation in distraction lengthening. A unique phenomenon called the “law of diminishing returns” whereby decreasing gains in spinal lengthening is observed have been reported with the MCGR. Divergence between the intended lengthening input and achieved lengthening has been reported with distraction [25, 26]. Reductions in lengthening may be due to reduced distraction forces as the rod lengthens [27]. Poon et al [28] showed in a biomechanical study that by using an unbent MCGR, the maximum forces were 103.0, 98.8 and 95.0 N at 0, 25 and 40 mm of rod protrusion, respectively. Another concern is metallosis around the rod-anchor junction and extendable portion of the MCGR [29]. Teoh et al [29] suggested metallosis to be a result of a chronic inflammatory response to metal debris which is produced via rod pistoning or telescoping. The significance of this debris and long-term effects are concerning but still unknown.

Recently, the world’s first MCGR patient just undergone her final fusion surgery. It is thus timely to report this clinical case and our own course of understanding the MCGR through the first decade of its use. Specifically the complications of this case with developments in clinical practice and rod modifications are highlighted.

## Case report

**Fig. 1:**
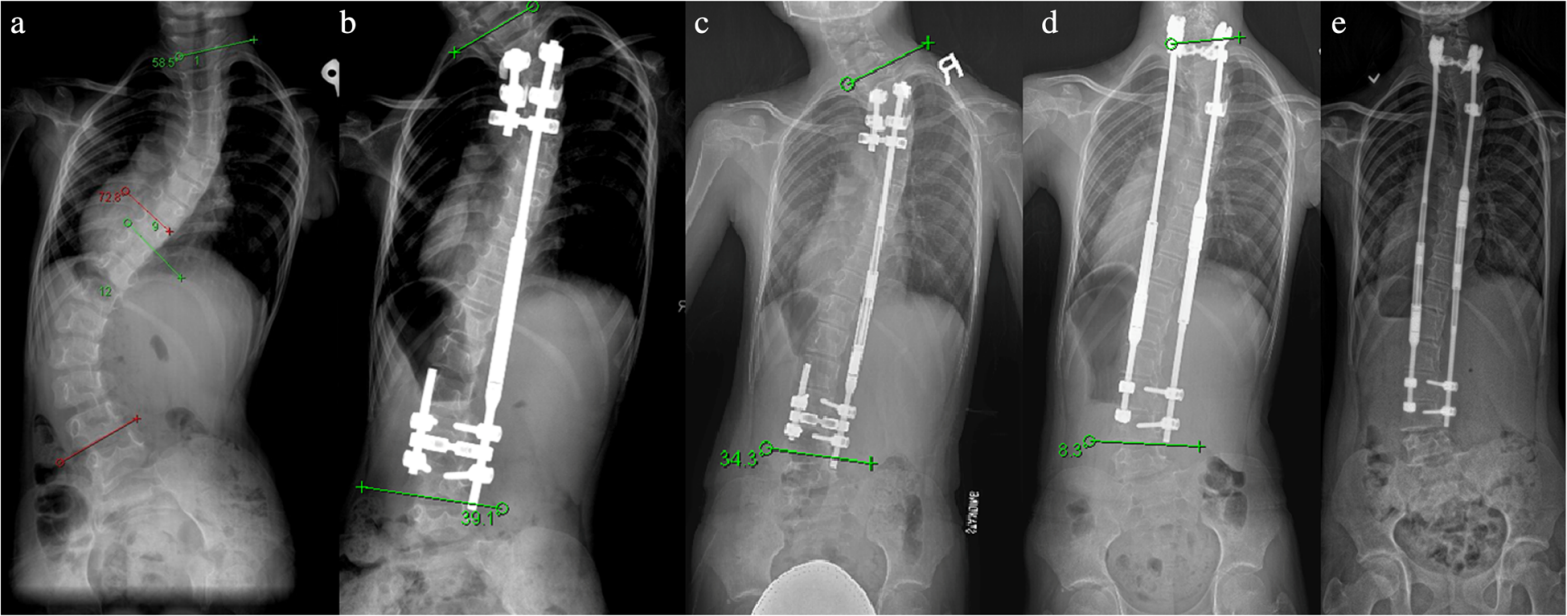
**a** Preoperative radiograph in 2009 showing 58.5 degrees from T1–9 and 72.8 degrees from T9-L4. **b** Single MCGR was anchored to T3–4 and L3–4. **c** The rod was distracted but overall coronal balance was suboptimal. **d** Dual MCGRs with extension of the proximal foundation to C7-T1 was performed. Note the enlarged portion with the magnet due to the internal keeper plate as compared to the same diameter magnet with the extendable portion of the rod in images **b** and **c**. **e** The rod distracted fully but developed add-on below

**Fig. 2:**
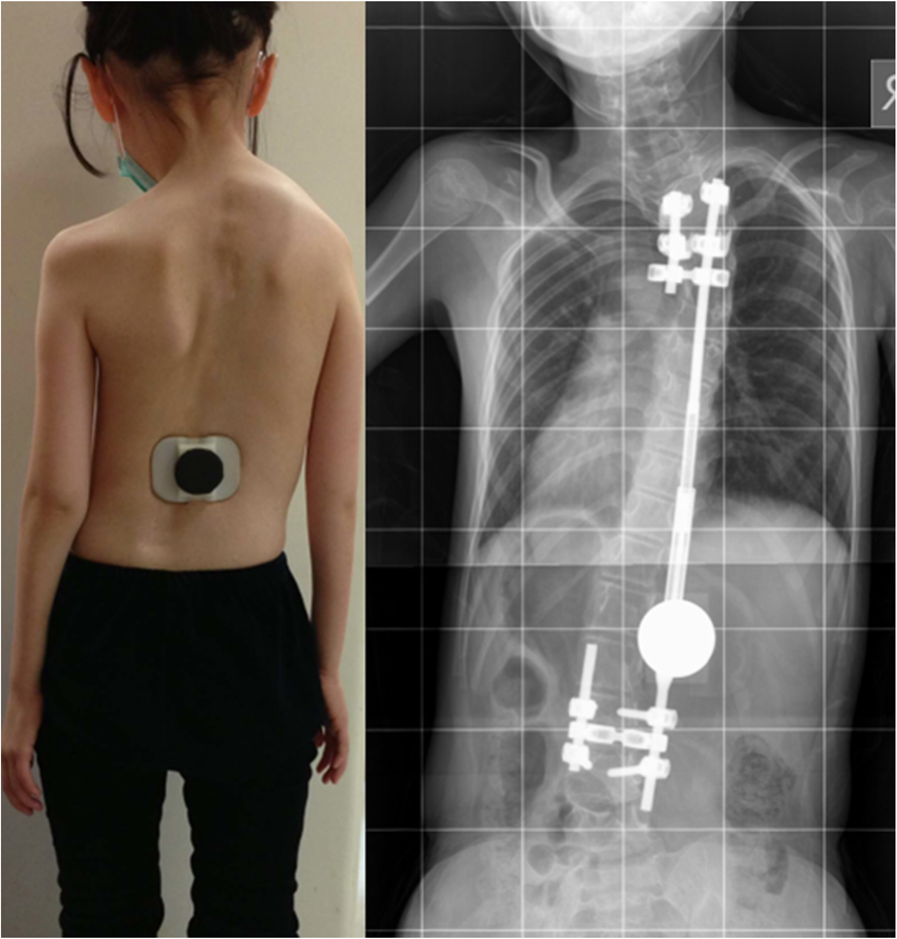
An external magnet used to prevent the 1st generation MCGR from losing distraction

**Fig. 3:**
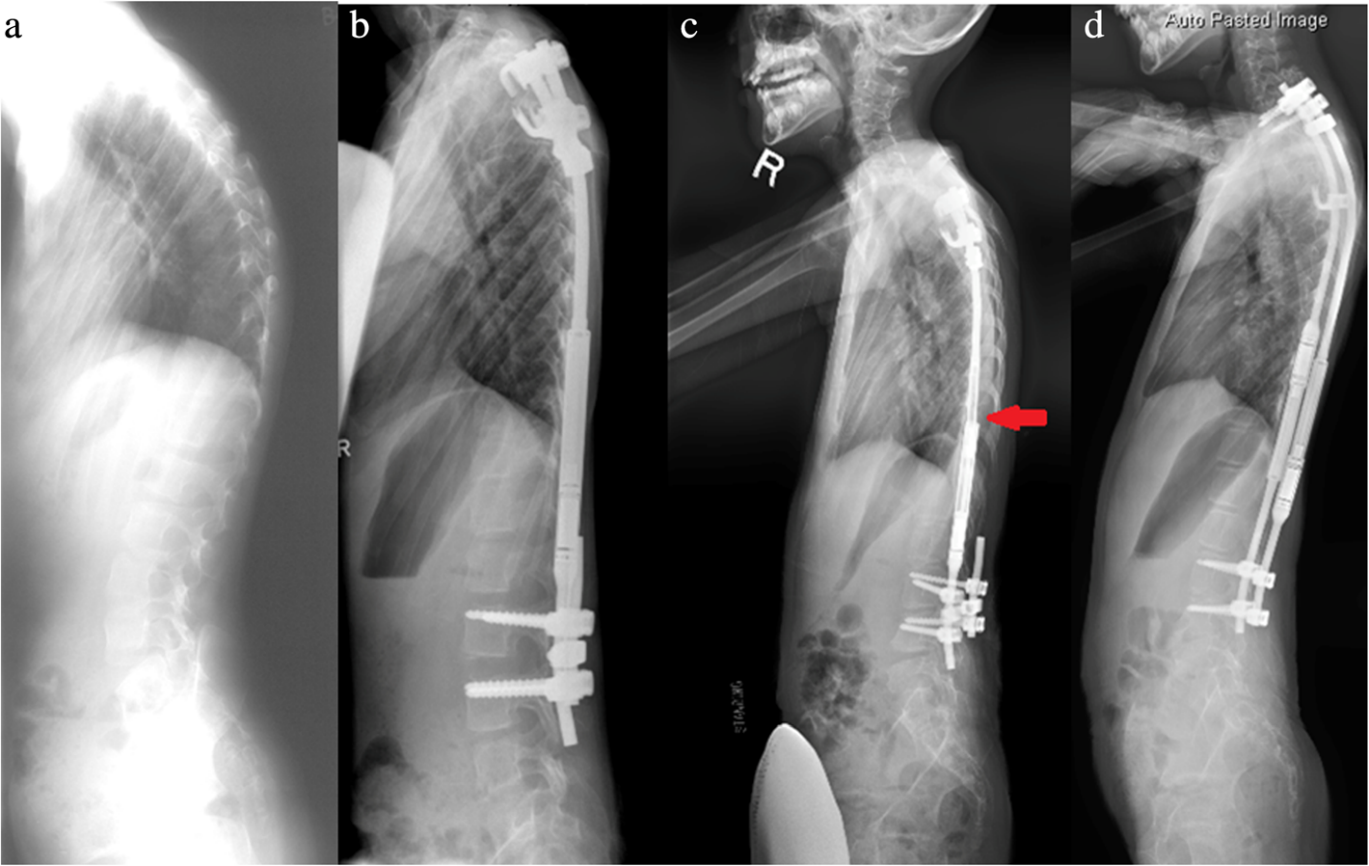
**a** Lateral radiographs preoperatively. **b** Initial single rod insertion with development of proximal junctional kyphosis after the revised rod. **c** A crooked rod sign in which the extendable portion of the rod is not inline with the actuator can be seen. **d** Extension to C7-T1 was performed

On 3rd November 2009, the first MCGR (Table 1) was implanted in a 5-year-old girl with Ehlers-Danlos Syndrome (type VI). This patient was born with generalized hypotonia and a flail right upper limb. She had a curve of 58.5 degrees at T1–9 and 72.8 degrees at T9-L4 with a single MCGR anchored at T3–4 and L3–4 (Fig. 1). An extra short rod was placed on the other side to facilitate any additional MCGR without changing the foundation. At postoperative 13 months, the MCGR failed to distract between lengthening episodes due to unrestricted turning of the internal magnet. The rod returned to the pre-distraction state at follow-up indicating a loss of distraction. The unrestricted turning was observed through internal testing by the developer. There was increasing truncal shift and shoulder elevation. An external magnet was placed outside the skin to prevent the magnet from turning back (Fig. 2). The rod was redesigned with an internal keeper plate added to prevent further loss of distraction (Fig. 1). The overall balance was suboptimal and after the rod was used up, she developed proximal junctional kyphosis (PJK) as well as a “crooked rod sign” (Fig. 3).

**Table 1:** Summary of surgeries

Age of patient	5 yr	9 yr	15 yr
Rod inserted	Single right standard MCGR	Dual MCGR: left standard, right offset	Fusion
Distraction episodes (rod lengthened)	26 distractions (37.6 mm)	28 distractions (left rod 46.4 mm, right rod 46.7 mm)	–
Complications related to implant	Loss of distraction, crooked rod sign, metallosis	Crooked rod sign, metallosis	Nil
Complications related to surgery	Proximal junctional kyphosis	Distal adding-on, autofusion	Nil

**Fig. 4:**
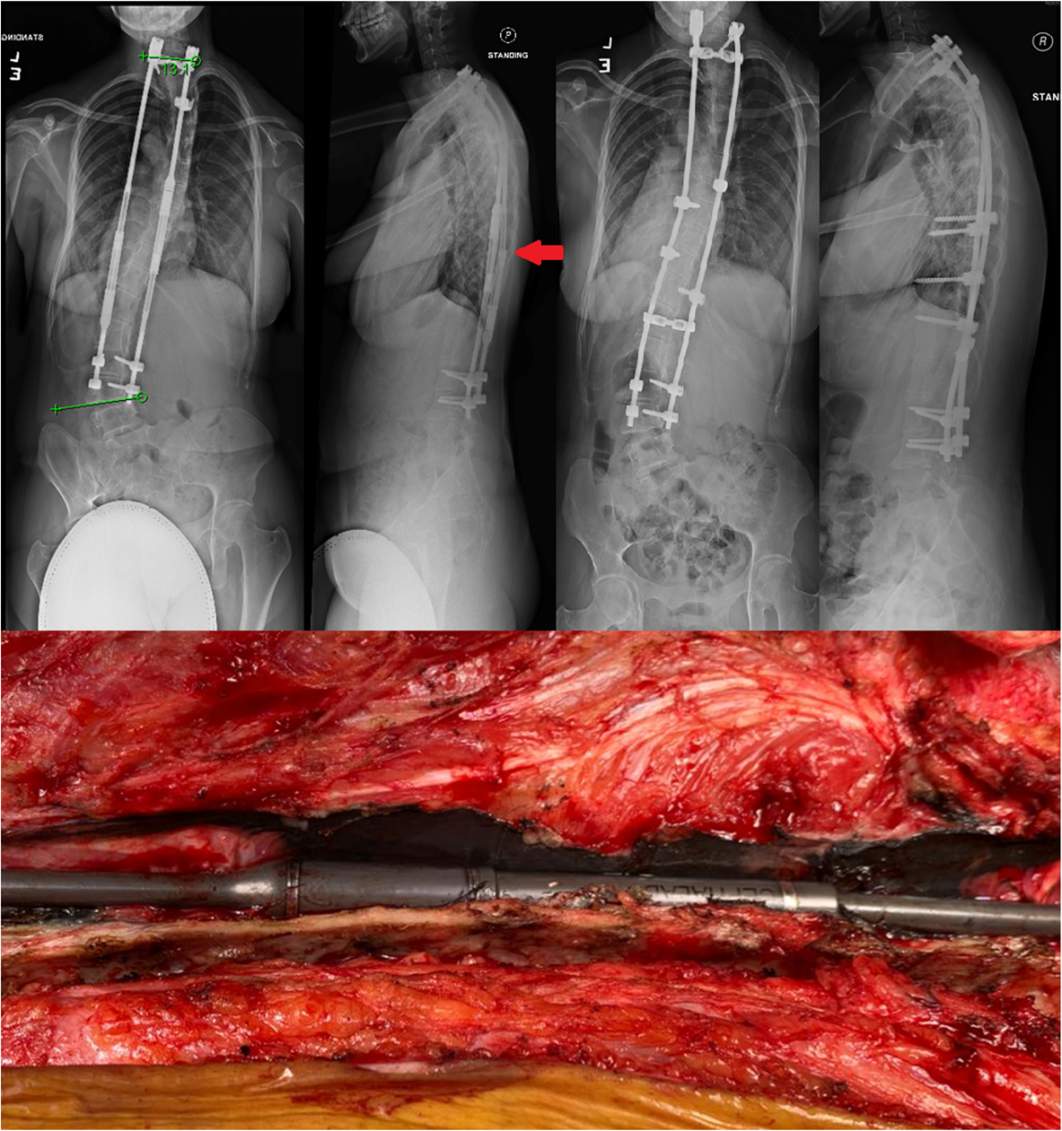
Pre-final surgery posteroanterior and lateral radiographs identified a crooked rod sign and autofusion of the lumbar spine (top left). Final fusion surgery from C7-L4 was performed (top right) leaving a residual tilt at L4-sacrum. Intraoperative photo showed extensive metallosis around the actuator and extendable portion of the rod (bottom)

At 9-years-old, a set of dual MCGRs with the new design were inserted with extension proximally to C7-T1. Her spinal balance improved and distractions continued. She subsequently developed adding-on [30] below (Fig. 1). At 15-years-old, the rods failed to distract with frequent rotor stalling. A “crooked rod sign” was again observed on radiographs (Fig. 4). No further distractions were possible. Autofusion was also observed in the lumbar spine. Final fusion surgery was performed from C7-L4 leaving a residual tilt below to avoid fusion to the pelvis as she was a candidate for the para-Olympics table tennis team and we wanted to maintain mobility. Gross metallosis observed around the actuator and extendable portion of the rod was debrided (Fig. 4).

**Fig. 5:**
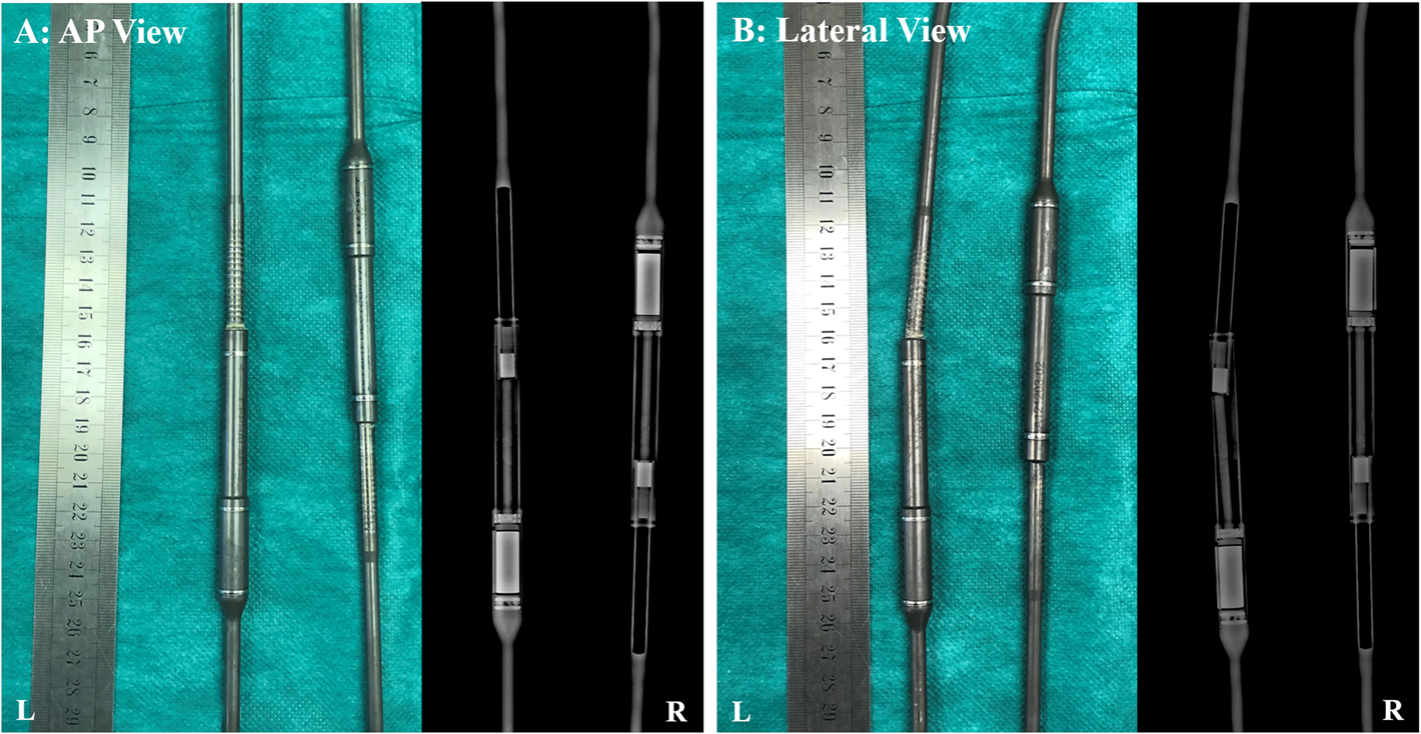
External examination of the final extracted MCGRs. **a** Both rods were aligned externally and under X-ray investigation in the AP view but some transverse wear marks could be seen on the piston rod portion. **b** On the lateral view, the “Crooked rod sign” was seen

**Fig. 6:**
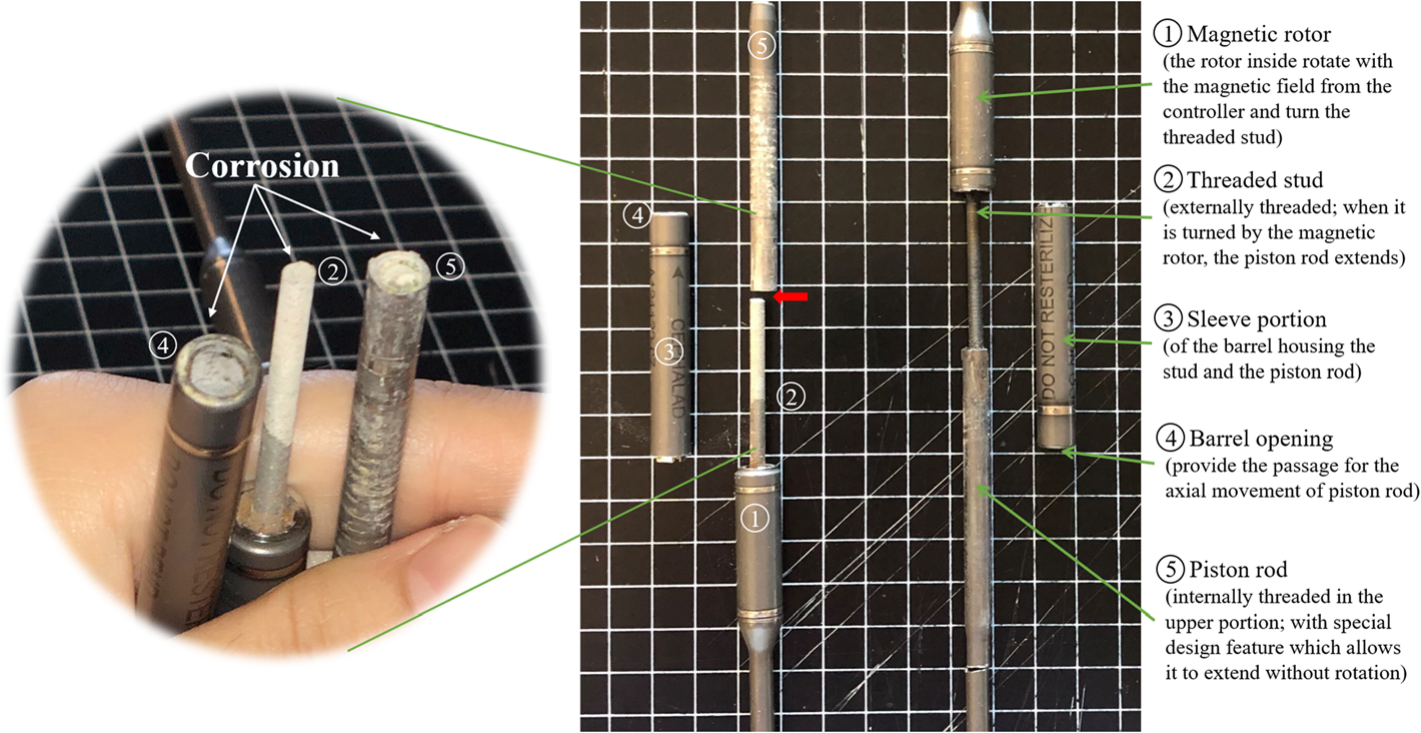
Internal examination of the distal extracted MCGRs. Post-dissection, the right rod was intact with the magnetic rotor (1), threaded stud (2), sleeve portion (3), barrel opening (4), and piston rod (5) aligned. The left rod had the threaded stud (2) fracture (red arrow) close to the barrel opening (4), with a propotion of the stud remained in the piston rod (5). Heavy corrosion was identified

The rods were extracted for visual inspection, X-ray examination and dissection (Fig. 5). On the external appearance, from the anteroposterior (AP) view, the two MCGRs were aligned. However, from the lateral view, the piston rod in the left MCGR showed a “crooked rod sign” close to the barrel opening. Dissection of the left rod revealed that the “crooked rod” radiographic sign was caused by fracture of the stud close to the barrel opening (Fig. 6). The rotor and stud could not drive the piston rod to extend due to this complete material failure. Part of the stud remained inside of the piston rod and the fracture site could have repetitive frictions caused by rod stalling during distraction sessions with magnet rotation. Corrosion could be seen at the stud fracture site and the barrel opening of the sleeve portion (Fig. 6).

**Fig. 7:**
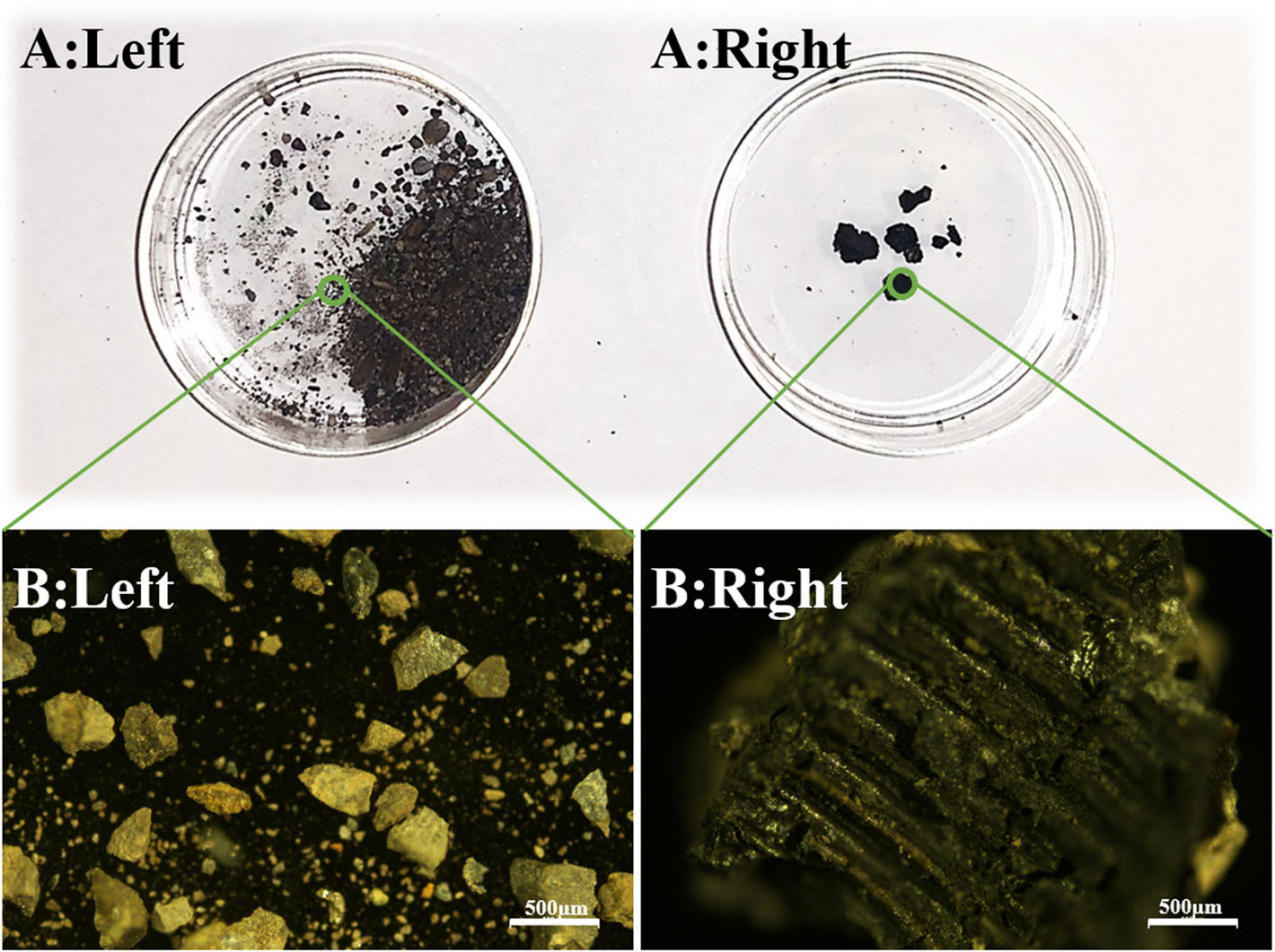
Debris from the MCGRs observed under light microscopy. **a** & **b** Left Morphologically, wear particles were seen for the left rod with fracture; **a** & **b** Right) whereas for the right rod, the debris was larger and had compressive screw markers

Additionally, the debris from inside of the sleeve was collected on petri dishes and observed under light microscopy (Fig. 7). Morphologically, wear particles were seen for the left rod with fracture (Fig. 7: A&B Left), whereas for the right rod, the debris was larger and had the appearance of screw thread tracks (Fig. 7: A&B Right). The concentrations of elements (mg/kg) in the sample were measured by inductively coupled plasma optical emission spectrometers (ICP-OES; Agilent 700 Series; Agilent Technologies, Inc.; US). The testing process followed the instructions from the manufacturer (https://www.agilent.com/cs/library/usermanuals). The ICP-OES revealed the elements from the debris contained both metal wear particles (Titanium, Aluminum, Vanadium, Neodymium) and human tissues (Calcium, Phosphate, Potassium, Sulfur, Sodium) (Table 2). For the left rod there was predominantly metal particles, whereas for the right rod, elements from human tissues were increased.

**Table 2:**
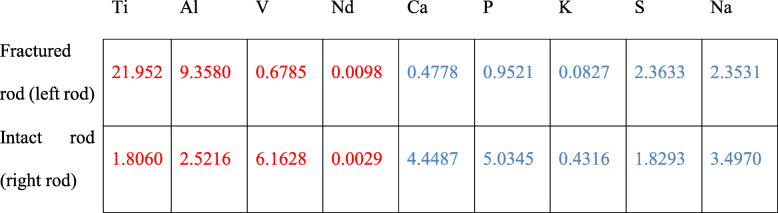
Inductively coupled plasma results of the powder from the dissected MCGRs

All listed in mg/kg.

Elements from the MCGR (red): Ti: Titanium; Al: Aluminum; V: Vanadium; Nd: Neodymium. Elements not from MCGR (blue): Ca: Calcium; P: Phosphate; K: Potassium; S: Sulfur; Na: Sodium.

The patient is now more than 2 years after the final fusion surgery with maintenance of the Cobb angle correction. The overall balance remains unchanged without any loosening of the implant.

## Discussion and conclusions

The MCGR and its surgical technique have gone through many iterations to solve various problems exhibited by this patient’s journey. The first MCGR or Phenix rod was developed by Arnaud Souberian, a French aeronautical engineer, in 2004 [12]. However, this rod fell out of use due to its large-sized internal magnet and the need for a permanent magnet to be placed on the skin. The widely adopted MCGR is the MAGEC® rod, initially developed by Ellipse Technologies, Inc. (Irvine, CA, USA) and subsequently acquired by NuVasive Inc. (San Diego, CA, USA) in 2016. The first tests of this novel technology was performed by Akbarnia et al [31] in Yucatan pigs which verified the mechanism of safe distractions using an external magnet. Subsequently, Cheung et al [13] described the very first experience with the MCGR in humans which supported its role in managing EOS. Since that initial experience, there has been six subsequent iterations or generations of the MCGR [16]. A “keeper plate” or stainless steel plate was inserted within the rod next to the magnet to prevent it from rotating on its own without the external magnet as illustrated in this patient. The “second generation” rod was introduced in 2010 with an increased rod shaft diameter from 9 mm to 10.5 mm to house this steel plate. The third generation rod was introduced in 2012 with a change from the previous pulsed welding method to a continuous welding method at the junction between the actuator and the rod shaft to reduce the risk of rod fractures. Since 2015, various other modifications have been made to allow a smaller sized actuator (70 mm) for smaller children, and reinforcement of the actuator pin [24]. The newest iteration of the MCGR is called MAGEC X which has since been withdrawn from use due to actuator cap dislodgement.

This report encompasses the entire journey of the first patient with MCGRs implanted till graduation. The encountered complications helped to improve the rod design. Loss of distraction was encountered between distraction sessions of the first single rod. The original rod design required the inclusion of a keeper plate which is a small stainless steel plate within the rod next to the magnet to prevent the rotor from rotating in the absence of the rotating magnetic field imposed by the external magnetic controller [16]. Although dual rods are generally preferred due to the large distraction forces and the possibility for differential correction [8, 13], only a single rod was possible due to her small physical size at the age of 5. The decision to use a claw construct may have led to PJK whereby no further kyphotic changes were observed after revision surgery with pedicle screws. The drawback to this was instrumentation into the cervical spine at final surgery. Another reason for the PJK could be flattening of the thoracic kyphosis by the actuator which extended into the thoracic spine for the early rods (Fig. 3). Building more proximal kyphosis was achievable when the patient was older and was physically big enough to accommodate longer rods for contouring. Often it is technically impossible to bend the rod much within a small distance between the proximal foundation and the actuator [32].

Besides the distraction failure due to the initial rod design, mechanical failures may occur such as actuator pin fracture [24] which has been improved by increased diameter of the pin with stronger material. However, breaks have still been reported with up to 21% in one series [33]. More importantly is the rotor stalling (slippage or clunking) which is more common in patients with increased body habitus such as older age and increased body mass during natural growth, as well as reduced distance between the two internal magnets due to cross-talk [27]. With MCGR wear, the stud threads in the rod may deform causing a failure of the internal distraction mechanism. Infiltration by body fluids and tissue may also occur which results in hastened wearing via corrosion of the threads as evidenced by the apparently intact right rod. However, close examination of the particles in the rod revealed screw compression marks and increased human tissue elements suggesting a seal fragmentation and failure. Further distractions are not achievable without revision surgery. This may be more important in those who routinely distract till stalling [34]. Radiologically, this appearance forms a “crooked rod sign” in which the extendable portion of the rod and the actuator are not in a parallel relationship [35]. Upon dissection of the final extracted rod however, we found a complete fracture of the threaded stud, which represents an extreme case of stud deformation and complete failure of rod extension, with metal wear particles accumulated inside the rod.

We speculate that there was corrosion inside of the barrel opening and wear marks on the piston rods was found to be associated with metallosis. Most MCGRs develop metallosis after prolonged use. This is observed around the rod-anchor junction and extendable portion of the MCGR in patients undergoing revision surgery [29]. This may be caused by increased wearing of the rod with creations of “growth marks” that occurs with each lengthening [36]. Up to 67% of patients at repeated surgery have been reported with such findings [37]. The long-term effect of the metallosis is unclear and is thus a concern as these are young patients who have yet to childbear. This may be a result of design defects and/or the O-ring seal failure (junction where the MCGR is extended) leading to metal-on-metal friction between the sleeve portion and the piston rod. Increased titanium, vanadium and neodymium concentrations in the muscle tissues surrounding the MCGR are found with chronic inflammations and phagocytotic black particles [38]. Titanium and vanadium are generated mainly at the barrel openings due to metal-on-metal contact, whereas the neodymium from the magnet rotor within the barrel is likely to be released from the barrel opening during distractions. The presence of neodymium proves that the O-ring seal has failed. Interestingly, there were more non-MCGR elements found in the intact rod as compared to the broken rod. We suspect that with a complete failure, further human tissue/fluid infiltration is limited while the intact rod continues to undergo cyclic loading with continuous wearing.

Best indications for surgery, advantages of dual rod constructs as well as signs of early complications and rod failure have been established. Despite a decade of use, aspects such as appropriate distraction intervals and technique, dealing with sagittal plane deformities and the long-term implications of metallosis still require further investigation. The best distraction frequency and amount of distractions per episode are important clinical questions as they influence lengthening outcomes. The Pediatric Spine Study Group is currently undergoing a multicenter randomized controlled trial in attempt to answer this question (ClinicalTrials.gov #NCT04058561). Modifying the surgical technique to maximize changes in the sagittal and axial plane is also important for further study. In addition, the effects of MCGR on lung function and vertebral remodeling with growth is unknown. Decision-making at graduation is also controversial. Whether rods should be removed or kept in-situ, or if fusion is needed are still debated.

## Data Availability

Not applicable as this is a case report.

## Abbreviations

AP: Anteroposterior
EOS: Early onset scoliosis
ICP-OES: Inductively coupled plasma-optical emission spectrometers
MCGR: Magnetically controlled growing rod
PJK: Proximal junctional kyphosis
TGR: Traditional growing rod

## References

[1] Akbarnia BA, Breakwell LM, Marks DS, McCarthy RE, Thompson AG, Canale SK (2008). Dual growing rod technique followed for three to eleven years until final fusion: the effect of frequency of lengthening. Spine (Phila Pa 1976).

[2] Akbarnia BA, Marks DS, Boachie-Adjei O, Thompson AG, Asher MA (2005). Dual growing rod technique for the treatment of progressive early-onset scoliosis: a multicenter study. Spine (Phila Pa 1976).

[3] Winter RB, Moe JH, Lonstein JE (1984). Posterior spinal arthrodesis for congenital scoliosis. An analysis of the cases of two hundred and ninety patients, five to nineteen years old. J Bone Joint Surg Am.

[4] Elsebai HB, Yazici M, Thompson GH, Emans JB, Skaggs DL, Crawford AH (2011). Safety and efficacy of growing rod technique for pediatric congenital spinal deformities. J Pediatr Orthop.

[5] Sponseller PD, Thompson GH, Akbarnia BA, Glait SA, Asher MA, Emans JB (2009). Growing rods for infantile scoliosis in Marfan syndrome. Spine (Phila Pa 1976).

[6] Sponseller PD, Yazici M, Demetracopoulos C, Emans JB (2007). Evidence basis for management of spine and chest wall deformities in children. Spine (Phila Pa 1976).

[7] Thompson GH, Akbarnia BA, Campbell RM (2007). Growing rod techniques in early-onset scoliosis. J Pediatr Orthop.

[8] Thompson GH, Akbarnia BA, Kostial P, Poe-Kochert C, Armstrong DG, Roh J (2005). Comparison of single and dual growing rod techniques followed through definitive surgery: a preliminary study. Spine (Phila Pa 1976).

[9] Akbarnia BA, Emans JB (2010). Complications of growth-sparing surgery in early onset scoliosis. Spine (Phila Pa 1976).

[10] Obid P, Yiu K, Cheung K, Kwan K, Ruf M, Cheung JPY. Magnetically controlled growing rods in early onset scoliosis: radiological results, outcome, and complications in a series of 22 patients. Arch Orthop Trauma Surg. 2020. 10.1007/s00402-020-03518-z. 10.1007/s00402-020-03518-z 32556642

[11] Cheung JPY, Cheung PWH, Cheung KMC (2020). The effect of magnetically controlled growing rods on three-dimensional changes in deformity correction. Spine Deform.

[12] Wick JM, Konze J (2012). A magnetic approach to treating progressive early-onset scoliosis. AORN J.

[13] Cheung KM, Cheung JP, Samartzis D, Mak KC, Wong YW, Cheung WY (2012). Magnetically controlled growing rods for severe spinal curvature in young children: a prospective case series. Lancet..

[14] Bess S, Akbarnia BA, Thompson GH, Sponseller PD, Shah SA, El Sebaie H (2010). Complications of growing-rod treatment for early-onset scoliosis: analysis of one hundred and forty patients. J Bone Joint Surg Am.

[15] Sankar WN, Skaggs DL, Yazici M, Johnston CE, Shah SA, Javidan P (2011). Lengthening of dual growing rods and the law of diminishing returns. Spine (Phila Pa 1976).

[16] Cheung JP, Cahill P, Yaszay B, Akbarnia BA, Cheung KM (2015). Special article: update on the magnetically controlled growing rod: tips and pitfalls. J Orthop Surg (Hong Kong).

[17] Samartzis D, Cheung JP, Rajasekaran S, Kawaguchi Y, Acharya S, Kawakami M (2016). Is lumbar facet joint tropism developmental or secondary to degeneration? An international, large-scale multicenter study by the AOSpine Asia Pacific research collaboration consortium. Scoliosis Spinal Disord.

[18] Kwan KYH, Cheung JPY, Yiu KKL, Cheung KMC (2018). Ten year follow-up of Jarcho-Levin syndrome with thoracic insufficiency treated by VEPTR and MCGR VEPTR hybrid. Eur Spine J.

[19] Cheung JP, Samartzis D, Cheung KM (2014). A novel approach to gradual correction of severe spinal deformity in a pediatric patient using the magnetically-controlled growing rod. Spine J.

[20] Welborn MC, Krajbich JI, D'Amato C (2019). Use of magnetic spinal growth rods (MCGR) with and without preoperative halo-gravity traction (HGT) for the treatment of severe early-onset scoliosis (EOS). J Pediatr Orthop.

[21] Charroin C, Abelin-Genevois K, Cunin V, Berthiller J, Constant H, Kohler R (2014). Direct costs associated with the management of progressive early onset scoliosis: estimations based on gold standard technique or with magnetically controlled growing rods. Orthop Traumatol Surg Res.

[22] Wong CKH, Cheung JPY, Cheung PWH, Lam CLK, Cheung KMC (2017). Traditional growing rod versus magnetically controlled growing rod for treatment of early onset scoliosis: cost analysis from implantation till skeletal maturity. J Orthop Surg (Hong Kong).

[23] Kwan KYH, Alanay A, Yazici M, Demirkiran G, Helenius I, Nnadi C (2017). Unplanned reoperations in magnetically controlled growing rod surgery for early onset scoliosis with a minimum of two-year follow-up. Spine (Phila Pa 1976).

[24] Jones CS, Stokes OM, Patel SB, Clarke AJ, Hutton M (2016). Actuator pin fracture in magnetically controlled growing rods: two cases. Spine J.

[25] Cobanoglu M, Shah SA, Gabos P, Rogers K, Yorgova P, Neiss G (2019). Comparison of intended lengthening of magnetically controlled growing rods: ultrasound versus X-ray. J Pediatr Orthop.

[26] Gilday SE, Schwartz MS, Bylski-Austrow DI, Glos DL, Schultz L, O'Hara S (2018). Observed length increases of magnetically controlled growing rods are lower than programmed. J Pediatr Orthop.

[27] Cheung JPY, Yiu KKL, Samartzis D, Kwan K, Tan BB, Cheung KMC (2018). Rod lengthening with the magnetically controlled growing rod: factors influencing rod slippage and reduced gains during distractions. Spine (Phila Pa 1976).

[28] Poon S, Spencer HT, Fayssoux RS, Sever R, Cho RH (2018). Maximal force generated by magnetically controlled growing rods decreases with rod lengthening. Spine Deform.

[29] Teoh KH, von Ruhland C, Evans SL, James SH, Jones A, Howes J (2016). Metallosis following implantation of magnetically controlled growing rods in the treatment of scoliosis: a case series. Bone Joint J.

[30] Wang Y, Hansen ES, Hoy K, Wu C, Bunger CE (2011). Distal adding-on phenomenon in Lenke 1A scoliosis: risk factor identification and treatment strategy comparison. Spine (Phila Pa 1976).

[31] Akbarnia BA, Mundis GM, Salari P, Yaszay B, Pawelek JB (2012). Innovation in growing rod technique: a study of safety and efficacy of a magnetically controlled growing rod in a porcine model. Spine (Phila Pa 1976).

[32] Cheung JPY, Cheung KM (2019). Current status of the magnetically controlled growing rod in treatment of early-onset scoliosis: what we know after a decade of experience. J Orthop Surg (Hong Kong).

[33] Joyce TJ, Smith SL, Kandemir G, Rushton PRP, Fender D, Bowey AJ (2020). The NuVasive MAGEC rod urgent field safety notice concerning locking pin fracture: how does data from an independent explant center compare?. Spine (Phila Pa 1976).

[34] Dahl B, Dragsted C, Ohrt-Nissen S, Andersen T, Gehrchen M (2018). Use of a distraction-to-stall lengthening procedure in magnetically controlled growing rods: a single-center cohort study. J Orthop Surg (Hong Kong).

[35] Cheung JPY, Zhang T, Bow C, Kwan K, Sze KY, Cheung KMC (2020). The crooked rod sign: a new radiological sign to detect deformed threads in the distraction mechanism of magnetically controlled growing rods and a mode of distraction failure. Spine (Phila Pa 1976).

[36] Joyce TJ, Smith SL, Rushton PRP, Bowey AJ, Gibson MJ (2018). Analysis of explanted magnetically controlled growing rods from seven UK spinal centers. Spine (Phila Pa 1976).

[37] Cheung JPY, Yiu K, Kwan K, Cheung KMC (2019). Mean 6-year follow-up of magnetically controlled growing rod patients with early onset scoliosis: a glimpse of what happens to graduates. Neurosurgery..

[38] Zhang T, Sze KY, Peng ZW, Cheung KMC, Lui YF, Wong YW (2020). Systematic investigation of metallosis associated with magnetically controlled growing rod implantation for early-onset scoliosis. Bone Joint J.

